# Growth of Porous Ag@AuCu Trimetal Nanoplates Assisted by Self-Assembly

**DOI:** 10.3390/nano10112207

**Published:** 2020-11-05

**Authors:** Wan-Cheng Zhang, Meng-Dai Luoshan, Peng-Fei Wang, Chu-Yun Huang, Qu-Quan Wang, Si-Jing Ding, Li Zhou

**Affiliations:** 1 School of Science, Hubei University of Technology, Wuhan 430068, China; wanchengzhang.nano@gmail.com (W.-C.Z.); Luosmd@hbut.edu.cn (M.-D.L.); chuyunh@163.com (C.-Y.H.); 2 Key Laboratory of Artificial Micro- and Nano-structures of the Ministry of Education, School of Physics and Technology, Wuhan University, Wuhan 430072, China; pfwang__opt@126.com (P.-F.W.); qqwang@whu.edu.cn (Q.-Q.W.); 3 School of Mathematics and Physics, China University of Geosciences, Wuhan 430074, China

**Keywords:** trimetal, self-assemble, nanoplates, AuCu alloy

## Abstract

The self-assembly process of metal nanoparticles has aroused wide attention due to its low cost and simplicity. However, most of the recently reported self-assembly systems only involve two or fewer metals. Herein, we first report a successful synthesis of self-assembled Ag@AuCu trimetal nanoplates in aqueous solution. The building blocks of multibranched AuCu alloy nanocrystals were first synthesized by a chemical reduction method. The growth of Ag onto the AuCu nanocrystals in the presence of hexadecyltrimethylammonium chloride (CTAC) induces a self-assembly process and formation of Ag@AuCu trimetal nanoplates. These nanoplates with an average side length of over 2 μm show a porous morphology and a very clear boundary with the branches of the as-prepared AuCu alloy nanocrystals extending out. The shape and density of the Ag@AuCu trimetal nanoplates can be controlled by changing the reaction time and the concentration of silver nitrate. The as-assembled Ag@AuCu nanoplates are expected to have the potential for wide-ranging applications in surface-enhanced Raman scattering (SERS) and catalysis owing to their unique structures.

## 1. Introduction

Metal nanostructures, such as AuAg, AuPt, and AuCu bimetal nanocrystals, have attracted extensive attention for decades owing to their outstanding applications ranging from plasmonics to catalysis [1,2,3,4,5,6,7]. Apart from the synthesis of metal nanocrystals with various configurations such as core–shell structures [8,9,10,11,12] and alloy structures [13,14,15,16,17,18], researchers have found that self-assembling metal nanocrystals into some unique structures can bring a series of fascinating properties. The self-assemblies of nanostructures also have many advantages, such as high efficiency, low cost, and accurate shape control, which make them ideal candidates for surface-enhanced Raman scattering (SERS) and sensing applications [19,20,21,22,23,24,25].

There are four main self-assembly methods: (i) assembled by electrostatic interaction (i.e., hydrogen bonding or covalent bonding) [26,27]; (ii) directed by templates such as carbon nanotubes [28,29], metal–organic frameworks [30], silica nanofibers [31], and graphene sheets [32]; (iii) based on biomolecules including DNA and proteins (e.g., viral protein) [33,34]; and (iv) coupled by the interaction between functional groups of copolymers in selective solvents [35,36]. The droplet evaporation method, which contains three main forces (i.e., van der Waals force, depletion force, and electrostatic force), is also a popular way to obtain self-assembled nanostructures [37,38,39,40]. So far, many unique self-assembled nanostructures have been synthesized, such as supercrystals [41,42,43,44,45], superlattices [46,47,48,49], dimers [11,50], rings [34,51], and so on [52,53,54,55]. Due to the charming optical properties of surface plasmon resonance (SPR) for gold and silver [56,57,58,59], the self-assembled nanostructures consisting of Au and Ag nanocrystals are the most reported. Wang and coworkers have performed an impressive synthesis of ordered superstructures assembled from differently shaped Au nanostructures (including Au nanorods, Au polyhedral, Au nanocubes, and Au bipyramids) using a droplet evaporation method [38]. Yang and coworkers have successfully synthesized the self-assembled Ag polyhedron superlattices with the densest packing [47]. A staircase superstructure was obtained by the self-assembly of Au@Ag core–shell nanocuboids using a two-step droplet evaporation method [40]. However, at present, most of the self-assembled systems only involve two or fewer metals, while the self-assembly phenomena of triple metals are rarely reported. Although self-assembly is a complex process involving multiple force interactions (Coulomb force, van der Waals force, capillary force, hydrophobic force, hydrogen-bonding interactions, etc. [44,60,61,62]) and the assembled system becomes more complex when more materials are involved, the self-assembly process is still worth studying due to its inspiring prospect, not only for metal materials but also for molecular materials [63].

In this study, we developed a successful synthesis of Ag@AuCu trigonal and hexagonal nanoplates assisted by self-assembly. By growing Ag on the AuCu alloy nanocrystals, a fascinating self-assembly phenomenon was observed in the presence of cationic surfactant (hexadecyltrimethylammonium chloride, i.e., CTAC). The self-assembly of AuCu alloy nanocrystals accompanied with the deposition, growth, and ripening of Ag leads to a formation of Ag@AuCu trimetal nanoplates. We further investigated the morphology controlling of the Ag@AuCu nanoplates by changing the concentration of AgNO_3_ and the reaction time. The as-synthesized nanoplates are expected to have the potential for wide-ranging surface-sensitive applications, such as catalysis, SPR, and SERS due to their unique structures.

## 2. Experimental

### 2.1. Materials

Ascorbic acid (AA, 99.7%), silver nitrate (AgNO_3_, 99.8%), sodium hydroxide (NaOH, 96.0%), cupric chloride dihydrate (CuCl_2_·2H_2_O, 99.0%), chloroauric acid (HAuCl_4_·4H_2_O, 99.9%), and glucose (α and β form) were purchased from Sinopharm Chemical Reagent Co., Ltd. (Shanghai, China). Hexadecylamine (HDA, 90.0%) was obtained from Aladdin Industrial Inc. (Shanghai, China). Hexadecyltrimethylammonium chloride (CTAC, 99.0%) was purchased from Sigma-Aldrich (USA). Deionized water with a resistivity of 18.25 MΩ·cm was used in all preparations.

### 2.2. Synthesis of AuCu Alloy Nanocrystals

AuCu alloy nanocrystals were prepared by a chemical reduction method. At first, 242.5 mg of HDA, 19.4 mL of deionized water, 0.9 mL of CuCl_2_ aqueous solution (100 mM), 3.2 mL of HAuCl_4_ aqueous solution (50 mM) and 1.5 mL glucose aqueous solution (1 M) were added into a 50 mL test tube at room temperature in sequence (the Au:Cu atomic ratio of 1.78). The whole capped solution was then magnetically stirred for 24 h at room temperature. After that, the test tube was transferred into an oil bath and heated at 100 °C for 40 min under magnetic stirring. As the reaction was completed, the color of this solution was changed from kelly green to black. For electron microscope characterization, the as-prepared product was centrifugated at 10,000 rpm for 8 min and washed with water three times and ethanol twice to remove excess precursor, HDA, and glucose. Finally, the washed samples were redispersed into 5 mL of deionized water.

### 2.3. Synthesis of Ag@AuCu Trigonal and Hexagonal Nanoplates

First, 3.5 mL of the as-prepared solution of AuCu alloy nanocrystals was centrifugated at 10,000 rpm for 8 min and redispersed into 70 mL of aqueous CTAC solution (80 mM). Next, 56 mL of the solution was divided into eight aliquots. After that, 0.01, 0.05, 0.1, 0.2, 0.5, 0.7, 1.0, and 2.0 mL of AgNO_3_ (0.01 M) were subsequently added into the eight aliquots (7 mL), respectively. Finally, 300 μL of ascorbic acid aqueous solution (0.1 M) and 50 μL of aqueous NaOH solution (2 M) were added into each aliquot. The color of the solutions was changed from black to deep brown. The resultant solutions were undisturbed and kept in an isothermal oven at 35 °C overnight (16 h).

### 2.4. Characterizations

Transmission electron microscopy (TEM) and high resolution TEM (HRTEM) images were recorded using a JEOL 2010 HT (JEOL Ltd., Tokyo, Japan) and a JEOL 2010 FET transmission electron microscope (JEOL Ltd., Tokyo, Japan) operated at 200 kV, respectively. The absorption spectra were tested by a UV–vis–NIR spectrophotometry (Cary 5000, Varian, America). Energy dispersive spectroscopy (EDS) analysis was performed on an EDS incorporated in the TEM. X-ray diffraction (XRD) pattern was obtained via a Rigaku SmartLab X-ray diffractometer (Rigaku, Tokyo, Japan) using Cu Kα irradiation (*λ* = 0.15418 nm).

## 3. Results and Discussion

### 3.1. Growth of Multibranched AuCu Alloy Nanocrystals

**Figure 1:**
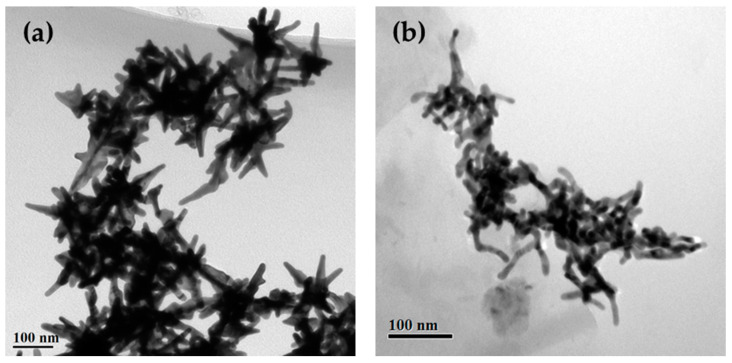
TEM images of AuCu nanocrystals with Au:Cu atom ratios of (**a**) 1.78 and (**b**) 0.33.

The chemical synthesis of gold-copper alloy nanocrystals uses CuCl_2_ and HAuCl_4_ as precursors, HDA as a capping agent, and glucose as a reductant. HDA in combination with glucose has been proved to produce Cu nanocrystals with controlled shapes in relatively large quantities and with high purity [64]. To begin with, aqueous solutions of CuCl_2_, HAuCl_4_, and glucose were added into a test tube containing a mixture of HDA and deionized water. After being capped, the test tube holding the resultant solution was magnetically stirred at room temperature for 24 h and then transferred into an oil bath heated at 100 °C for 40 min under magnetic stirring. Along with the proceeding of the reaction, the solution color was changed from kelly green to black, indicating the formation and morphological evolution of AuCu alloy nanocrystlas. Based on our previous work [18], multibranched AuCu alloy nanocrystals could be formed, and the morphological parameters, such as branch number and branch length, were determined by the atomic ratio of Cu in the precursors. The TEM images of the typical AuCu alloy nanocrystals with different Au:Cu atomic ratios (1.78 and 0.33) are shown in Figure 1. The high resolution TEM image of AuCu alloy nanocrystals with an Au:Cu atomic ratio of 1.78 was shown in Figure S1; the lattice spacings marked around the upper right corner and the left side of branch ends can all be indexed to {111} planes. The XRD pattern of AuCu nanocrystals shown in Figure S2 can be indexed as a face-centered cubic (fcc) structure, indicating the AuCu alloy phase. Multibranched nanostars (Figure 1a) and irregular multibranched nanocrystals (Figure 1b) can be observed in these images. The multibranched metal nanocrystals can provide a large area of active surfaces with plasmonic and catalytic hot spots, such as the sharp tips and inter-branch gap regions with highly localized field enhancement, as well as the high-index facets covered by atomic steps, edges, and kinks. We chose the multibranched AuCu alloy nanostars shown in Figure 1a as a substrate for the growth of Ag in producing a trimetal nanocrystal with potential plasmonic and catalytic properties.

### 3.2. Optical Properties of Ag@AuCu Nanostructures

**Figure 2:**
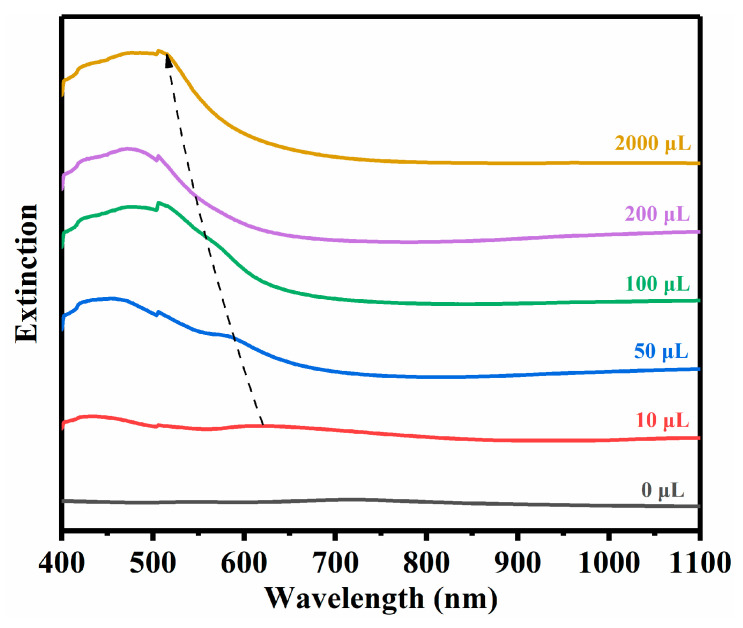
Extinction spectra of Ag@AuCu nanostructures (the Au:Cu atom ratio is 1.78) in aqueous solution. The numbers above the curves indicate the amounts of AgNO_3_.

By growing Ag onto the AuCu multibranched nanocrystals, we successfully synthesized the Ag@AuCu trigonal and hexagonal nanoplates assisted by self-assembly. The optical properties of the as-synthesized samples in aqueous solution were studied by the extinction spectra, as shown in Figure 2 and Figure S3. The extinction spectra of AuCu alloy nanocrystals with the Au:Cu atom ratios of 1.78 and 0.33 are shown in Figure S3. A transverse SPR (T-SPR) peak around 530 nm is observed, which is caused by the combination of Au and Cu [65,66]. For the case of Au:Cu atom ratio of 1.78, two SPR peaks can be clearly observed, similar to our previous report [18]. The intense and broad absorption of the AuCu nanocrystals with an Au:Cu atom ratio of 0.33 in the NIR region (Figure S3) may be attributed to the wide distribution of branch lengths [18]. Figure 2 shows the extinction spectra of synthesized Ag@AuCu nanostructures with different amounts of AgNO_3_. Two bands respectively near 420 nm and 620 nm appeared when 10 μL of AgNO_3_ was added. With increasing the amount of AgNO_3_, the band near 620 nm is blue-shifted and a broad band ranged from 400 nm to 550 nm is finally formed. This spectral evolution may be attributed to the coating of silver and the self-assembly of multibranched alloy nanocrystals.

### 3.3. Initial Formation of Ag@AuCu Trigonal Nanoplates Assisted by Self-Assembly

**Figure 3:**
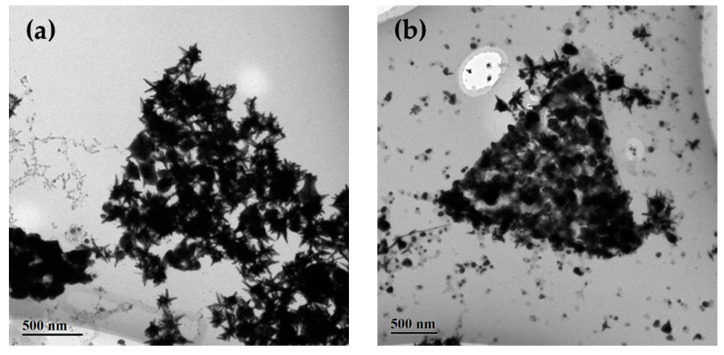
Initial formation of trigonal nanoplates of Ag@AuCu with a low concentration of AgNO_3_ (50 μL). (**a**) Embryonic form of a self-assembled Ag@AuCu trigonal nanoplate. (**b**) Initial formation of a trigonal nanoplate with a clear boundary. The average side length is 2.12 ± 0.04 μm.

To grow Ag on the as-prepared AuCu samples, the AuCu alloy nanocrystals were first redispersed into aqueous CTAC solution. Subsequently, AgNO_3_, ascorbic acid, and NaOH were added into the solution. As shown in Figure S4, the synthesized Ag nanoparticles through the reduction of Ag precursors tended to assemble. Figure S4a shows the assembly of two Ag nanoparticles to form a dimer. As the amount of Ag nanoparticles increased, they began to assemble into a trigonal structure (Figure S4b,c), indicating its high stability (Figure S4d). Similarly, following the deposition of Ag onto the multibranched AuCu nanocrystals, the formed Ag@AuCu trimetal nanocrystals showed a self-assembly behavior. An embryonic form of Ag@AuCu trigonal nanoplates with a low concentration of AgNO_3_ (50 μL) can be observed from the TEM images (Figure 3). A clear boundary of Ag@AuCu trigonal nanoplate with an average side length of 2.12 ± 0.04 μm appeared several hours later (Figure 3b), which indicated the initial formation of a trigonal nanoplate. Meanwhile, the growth of Ag could also be observed on the surface of multibranched AuCu nanocrystals, as there was a morphological change for the multibranched nanocrystals compared with their initial shape shown in Figure 1. The TEM image of a transitional form shown in Figure S5 shows the assembly and aggregation of AuCu multibranched nanocrystals and Ag nanoparticles. The EDS spectrum of the Ag@AuCu trigonal nanoplates is shown in Figure S6, and the elements of Ag, Au, and Cu can be clearly observed in the EDS spectrum.

A similar but much smaller structure called “porous 2D AuCu triangular nanoprism” has been reported and those as-synthesized triangular nanoprisms with large surface areas and high energy facets have been proved to obtain high electrocatalytic activity for the electrooxidation of ethylene glycol (EGOR) and glycerol (GOR) [67]. In this work, the addition of Ag endows the porous Ag@AuCu trimetal nanoplates with more desirable and active properties. Previous literature has shown many self-assembly phenomena of nanocrystals through solvent evaporation of a droplet or in bulk aqueous solution containing concentrated surfactant (e.g., CTAC) and nanocrystals [68,69,70,71]. The proposed driving force is the coordinated actions of layered micellar structures of surfactant molecules. The interactions of the surfactant molecules between adjacent nanoparticles arrange them in a stable three-dimensional structure. According to previous research, the self-assembly of nanocrystals is a slow process. The shape and feature of Ag@AuCu nanoplates are highly related to the concentration of AgNO_3_ and largely dependent on the reaction time.

### 3.4. Growth of Ag@AuCu Trigonal Nanoplates with a Moderate Concentration of AgNO_3_

**Figure 4:**
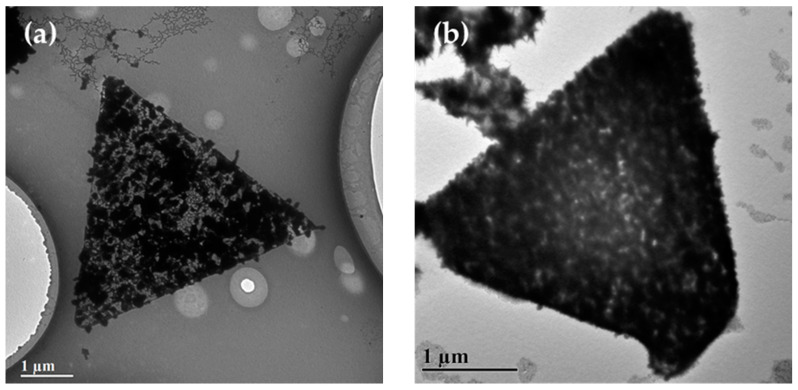
Formation and growth of Ag@AuCu trigonal nanoplates with a moderate concentration of AgNO_3_ (200 μL) at different reaction times. (**a**) A porous trigonal nanoplate of self-assembled Ag@AuCu nanocrystals with an average side length of 4.85 ± 0.1 μm. (**b**) A high-dense trigonal nanoplate with star-branches on the boundary. The average side length is 2.92 ± 0.1 μm.

As the addition amount of AgNO_3_ increased to 200 μL, a kind of porous trigonal nanoplate (Figure 4a) and a high-dense one (Figure 4b) were observed. The TEM images in Figure 4 were taken from the same sample but with different reaction times. The reaction time of the sample in Figure 4b is five days longer than that in Figure 4a. The porous and high-dense trigonal nanoplates represented different stages of the formation of Ag@AuCu trigonal nanoplates. Two processes played a role in the formation of the Ag@AuCu nanoplates: (1) self-assembly; (2) reduction and growth of Ag. As time passed, the complex interaction of various forces that drives the formation of the Ag@AuCu trigonal nanoplate would make it move towards a more stable nanostructure. For instance, a loose nanoplate would be shrunk to a dense one. That is why the high-dense nanoplates are always smaller than the porous ones. On the other hand, the self-assembly was accompanied by the reduction and growth of Ag. Ag atoms were deposited onto the AuCu nanocrystals or Ag nanoparticles were self-nucleated and grown. The deposition, growth, and ripening processes of Ag could partially fill the void space and connect the AuCu nanocrystals, making the assembled Ag@AuCu nanostructures denser and more stable. In Figure 4b, many star-branches extending out the boundary of the high-dense trigonal nanoplate can be observed. The porous structure is supposed to be maintained for the high-dense trigonal nanoplates. Highly branched and porous nanocrystals have a higher surface-to-volume ratio and rougher surface than isotropic nanocrystals, and nanostructures with branching arms on the surface often have the characteristic of a high refractive index [17]. The as-synthesized Ag@AuCu trigonal nanoplates may obtain the potential for wide-ranging applications in many fields and become ideal candidates for some surface-sensitive applications [72,73,74].

### 3.5. Growth of Ag@AuCu Hexagonal Nanoplates with a High Concentration of AgNO_3_

**Figure 5:**
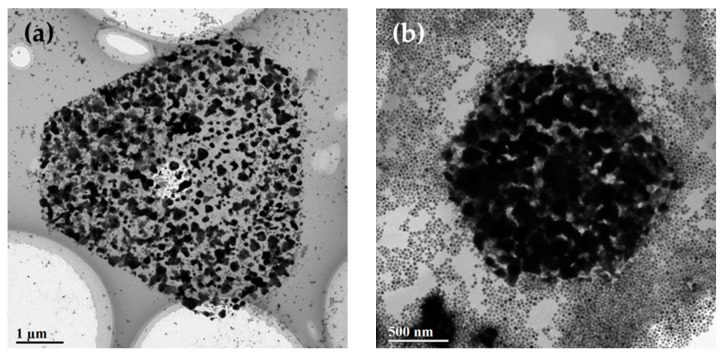
Formation and growth of Ag@AuCu hexagonal nanoplates with a high concentration of AgNO_3_ (2000 μL). (**a**) A porous hexagonal nanoplate of self-assembled Ag@AuCu nanocrystals. The average length of its long side and short side is 4.10 ±0.1 μm and 2.07 ± 0.1μm, respectively. (**b**) A high-dense hexagonal nanoplate.

When the addition amount of AgNO_3_ reached 2000 μL, the hexagonal Ag@AuCu nanoplates were observed in the product. Similarly, the loose and porous nanoplates (Figure 5a) as well as the high-dense nanoplates (Figure 5b) were both obtained. In this case, a large scale of Ag nanoparticles is observed in Figure 5, which is due to the high concentration of AgNO_3_ and the self-nucleation of Ag. As seen in Figure S7 and Figure S8, for some hexagonal nanoplates, the edge region is denser, whereas the interior region is relatively loose. We assume that the overgrowth of Ag is mainly located on the edges of nanoplates in the condition with a high concentration of AgNO_3_.

## 4. Discussion and Conclusions

**Figure 6:**
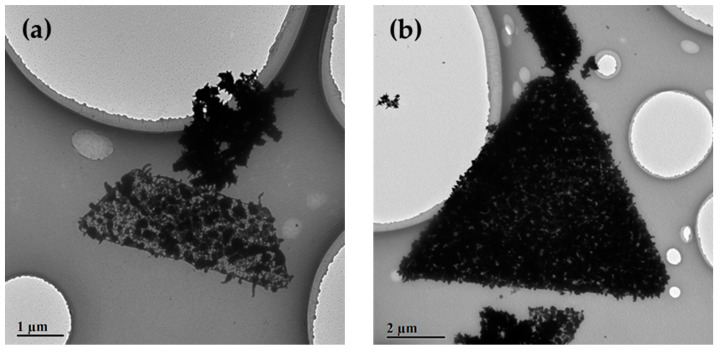
Complex nanostructures of Ag@AuCu trigonal nanoplates with a moderate concentration of AgNO_3_ (200 μL). (**a**) A cluster of Ag@AuCu nanostructures on a half trigonal nanoplate. (**b**) A nanowire of Ag@AuCu nanostructures connected to a trigonal nanoplate.

In this study, we also observed some interesting morphologies of the Ag@AuCu trimetal nanoplates. As shown in Figure 6a, the Ag@AuCu nanostructures clustered together on a trapezoidal nanoplate (half of a trigonal nanoplate or, in other words, an incomplete trigonal nanoplate). In Figure 6b, a giant self-assembled nanowire connected to the top of an Ag@AuCu trigonal nanoplate. Similar phenomena also took place on the Ag@AuCu hexagonal nanoplates (Figure S8), and several tiny trigonal nanoplates and hexagonal nanoplates were stacked on a huge hexagonal nanoplate. Although there were some incomplete reactions, the complete trigonal nanoplates with clear boundaries are the dominant product (~60%), as shown in Figure S9.

We have successfully synthesized the AuCu alloy multibranched nanocrystals using a chemical reduction method in aqueous solution. Au is a stable plasmonic metal and widely used in many applications. Cu is a low-cost plasmonic material; however, the synthesis of Cu nanocrystals needs to be further developed. The combination of Au and Cu has been reported to produce some interesting morphologies and lead to largely enhanced catalytic performances [17]. Here, another excellent plasmonic metal of Ag with low damping is integrated with AuCu alloy nanocrystals. During the process of growing Ag on the as-prepared AuCu alloy multibranched nanocrystals, some interesting self-assembly phenomena were observed. When the addition amount of AgNO_3_ was as low as 50 μL, owing to the assembly of AuCu alloy nanocrystals and the growth of Ag, the Ag@AuCu hybrid nanostructures clustered together and began to self-assemble into a trigonal nanoplate. An embryonic form of Ag@AuCu trigonal nanoplate was formed due to the coordinated actions of layered micellar structures of CTAC. Subsequently, the deposition, growth, and ripening processes of Ag are supposed to influence and stabilize the morphology of nanoplates. As the addition amount of AgNO_3_ increased to 200 μL, the increased amount of Ag growth made the morphology of the Ag@AuCu hybrid nanostructures more complete and more tunable. When we added 2000 μL AgNO_3_ into the AuCu alloy nanocrystals, the Ag@AuCu hybrid nanostructures tended to form hexagonal nanoplates rather than trigonal ones. As the knowledge of self-assembly processes at the nanoscale is not fully understood yet, only a brief discussion is put forward on the growth mechanism of Ag@AuCu trigonal nanoplates in this article. In another case, the Ag@AuCu trigonal nanoplates can also be observed when we grew Ag on the AuCu alloy nanocrystals with an Au:Cu atom ratio of 0.33 (Figure S10), confirming the universality of this method.

In conclusion, we report a method to grow a new type of trimetal Ag@AuCu trigonal and hexagonal nanoplates assisted by self-assembly. The morphology of Ag@AuCu nanoplates is highly related to the concentration of AgNO_3_ and the reaction time. We infer that the self-assembly and formation of porous Ag@AuCu nanoplates are caused by the coordinated actions of layered micellar structures of CTAC, as well as the accompanied deposition, growth, and ripening processes of Ag. As far as we know, this is the first report about such self-assembled trimetal nanoplates. The porous morphology and the extending branches from their edges are expected to bring some fascinating new features. These unique porous and trimetal nanoplates may have the potential for wide-ranging surface-sensitive applications.

## Supplementary Materials

The following are available online at https://www.mdpi.com/2079-4991/10/11/2207/s1, Figure S1: High resolution TEM image of AuCu alloy nanocrystals with an Au:Cu atomic ratio of 1.78; Figure S2: XRD patterns of AuCu nanocrystals and Ag@AuCu nanoplates. The XRD pattern of AuCu nanocrystals is similar to the pentacle AuCu nanocrystals reported in Reference [17]. As the lattice constant of Au and Ag is similar, the Au phase and Ag phase is unresolved in the XRD pattern. The XRD pattern is almost unchanged after the self-assembly and Ag deposition; Figure S3: Extinction spectra of AuCu alloy nanostructures with different Au:Cu atom ratios in aqueous solution; Figure S4: The initial self-assembly of Ag nanoparticles forming a triangular structure: (a–c) TEM images of Ag nanoparticles; (d) The self-assembly model of Ag nanoparticles, the triangular structure obtains higher stability; Figure S5: TEM image of the transitional form of Ag@AuCu trigonal nanoplates between Figure 3a,b with a low concentration of AgNO_3_ (50 μL); Figure S6: EDS spectrum of the Ag@AuCu trigonal nanoplates; Figure S7: TEM images of Ag@AuCu hexagonal nanoplates with a relatively empty interior, their average side lengths are (a) 2.36 ± 0.1 μm and (b) 1.48 ± 0.1 μm; Figure S8: TEM image of complex nanostructures of Ag@AuCu hexagonal nanoplates with a high concentration of AgNO3 (2000 μL), several tiny trigonal nanoplates and hexagonal nanoplates can be observed above a huge hexagonal nanoplate; Figure S9: TEM image and statistical data of Ag@AuCu trigonal nanoplates. The dashed triangles in (a) mark the Ag@AuCu nanoplates with completed trigonal shape. The histogram in (b) show the percentages of three kinds of Ag@AuCu nanostructures: completed—nanoplates with a clear trigonal boundary (both porous and solid nanoplates); incomplete—incomplete shapes, like wires and trapezoids; and irregular—nanostructures with irregular shapes; Figure S10: Growth of solid trigonal nanoplate of the Ag@AuCu nanostructures based on the AuCu alloy nanocrystals with an Au:Cu ratio of 0.33 using another method.
