# Supplementary material for: Growth of Porous Ag@AuCu Trimetal Nanoplates Assisted by Self-Assembly
Source: Nanomaterials (Basel). 2020 Nov 5;10(11):2207. doi: 10.3390/nano10112207 (PMC7694533; doi:10.3390/nano10112207)
Supplement: Supplementary file 1 [file nanomaterials-10-02207-s001.pdf]

nanomaterials

Supplementary Material

Growth of Porous Ag@AuCu Trimetal Nanoplates Assisted by Self-Assembly

Wan-Cheng Zhang [1,†], Meng-Dai Luoshan [1,2,†], Peng-Fei Wang [2], Chu-Yun Huang [1],

Qu-Quan Wang [2], Si-Jing Ding [3,*] and Li Zhou [2,*]

1 School of Science, Hubei University of Technology, Wuhan 430068, China; wanchengzhang.nano@gmail.com (W.-C.Z.); Luosmd@hbut.edu.cn (M.-D.L.); chuyunh@163.com (C.-Y.H.)
2 Key Laboratory of Artificial Micro- and Nano-structures of the Ministry of Education, School of Physics and Technology, Wuhan University, Wuhan 430072, China; pfwang__opt@126.com (P.-F.W.); qqwang@whu.edu.cn (Q.-Q.W.)
3 School of Mathematics and Physics, China University of Geosciences, Wuhan 430074, China.
* Correspondence: dingsijing@cug.edu.cn (S.-J.D.); zhouli@whu.edu.cn (L.Z.)
† These authors equally contributed to this work.

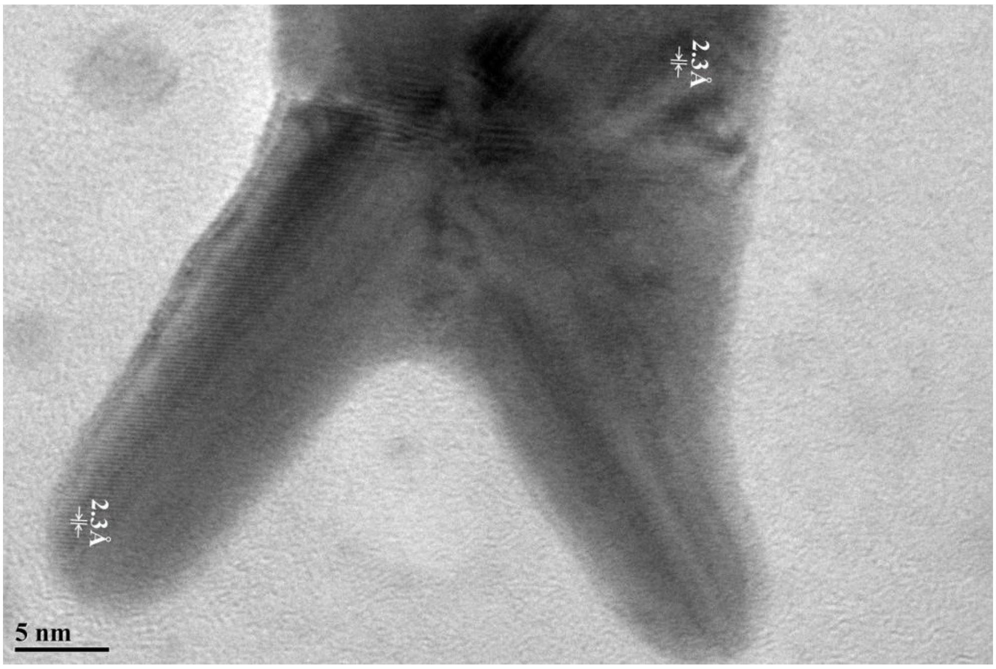

Figure S1. High resolution TEM image of AuCu alloy nanocrystals with an Au:Cu atomic ratio of 1.78.

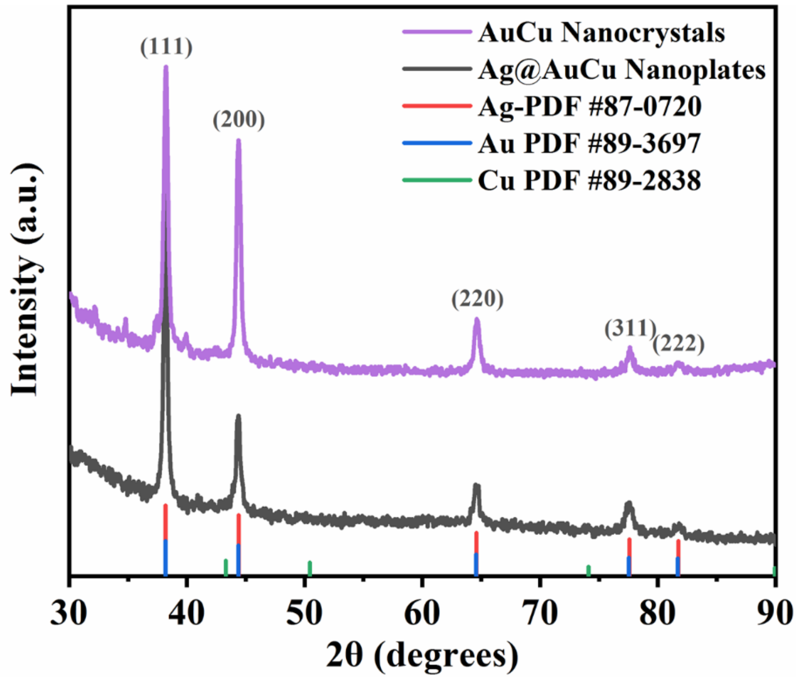

Figure S2. XRD patterns of AuCu nanocrystals and Ag@AuCu nanoplates. The XRD pattern of AuCu nanocrystals is similar with the pentacle AuCu nanocrystals reported in Ref [17]. As the lattice constant of Au and Ag is similar, the Au phase and Ag phase is unresolved in XRD pattern. The XRD pattern is almost unchaged after the self-assembly and Ag deposition.

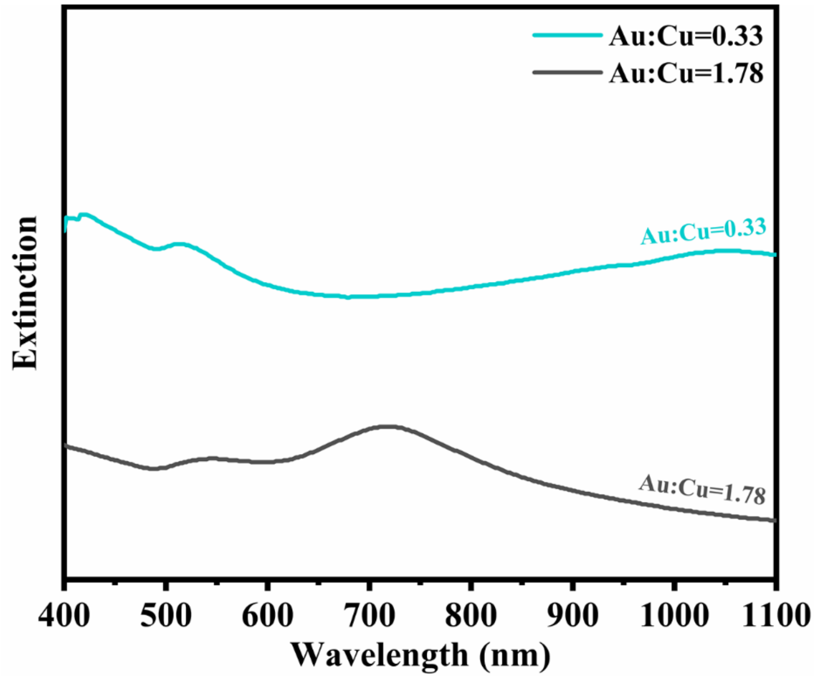

Figure S3. Extinction spectra of AuCu alloy nanostructures with different Au:Cu atom ratios in aqueous solution.

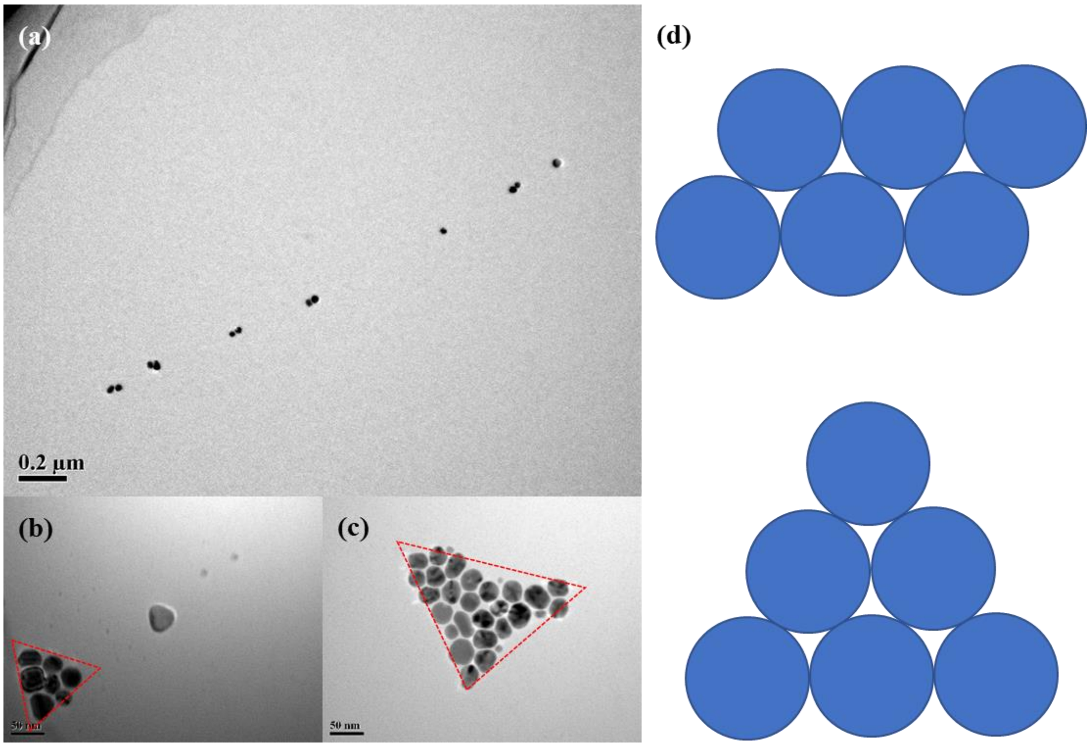

Figure S4. The initial self-assembly of Ag nanoparticles forming a triangular structure: (**a**–**c**) TEM images of Ag nanoparticles; (**d**) The self-assembly model of Ag nanoparticles, the triangular structure obtains higher stability.

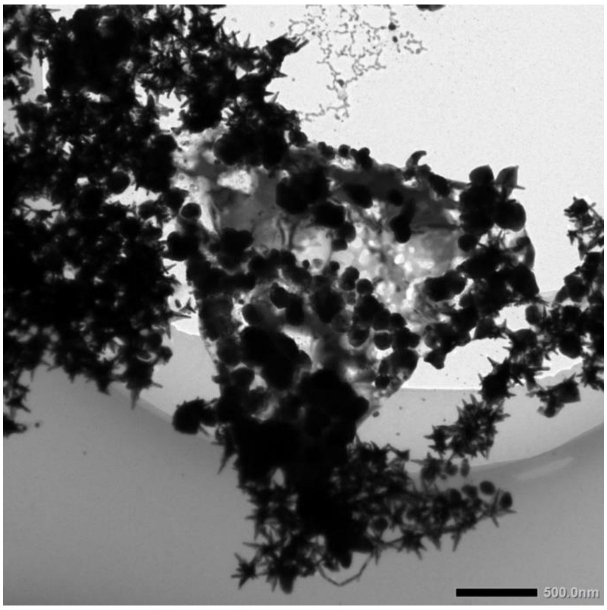

Figure S5. TEM image of the transitional form of Ag@AuCu trigonal nanoplates between Figure 3(a) and Figure 3(b) with a low concentration of $AgNO_3$ (50 μL).

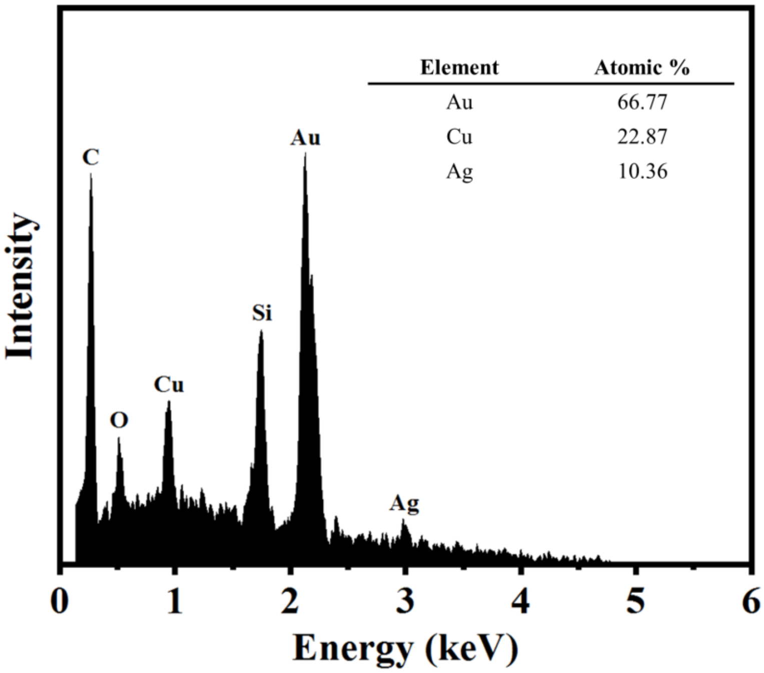

Figure S6. EDS spectrum of the Ag@AuCu trigonal nanoplates. The compositions of Au, Cu, and Ag are all observed.

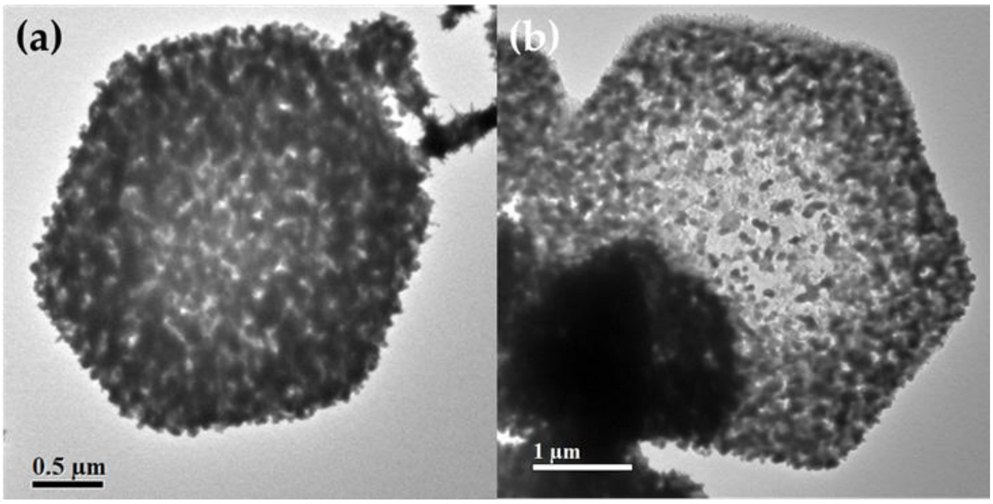

Figure S7. TEM images of Ag@AuCu hexagonal nanoplates with a relatively empty interior. Their average side lengths are (**a**) 2.36 ± 0.1 µm and (**b**) 1.48 ± 0.1 µm.

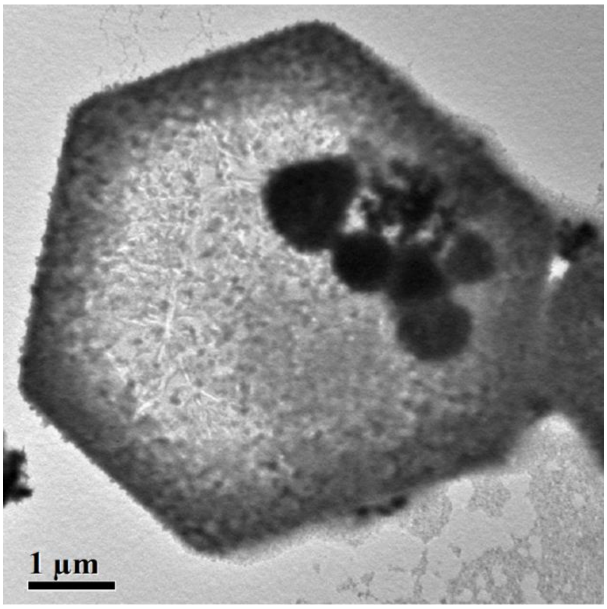

Figure S8. TEM image of Complex nanostructures of Ag@AuCu hexagonal nanoplate with a high concentration of $AgNO_3$ (2000 µL). Several tiny trigonal nanoplates and hexagonal nanoplates can be observed above a huge hexagonal nanoplate.

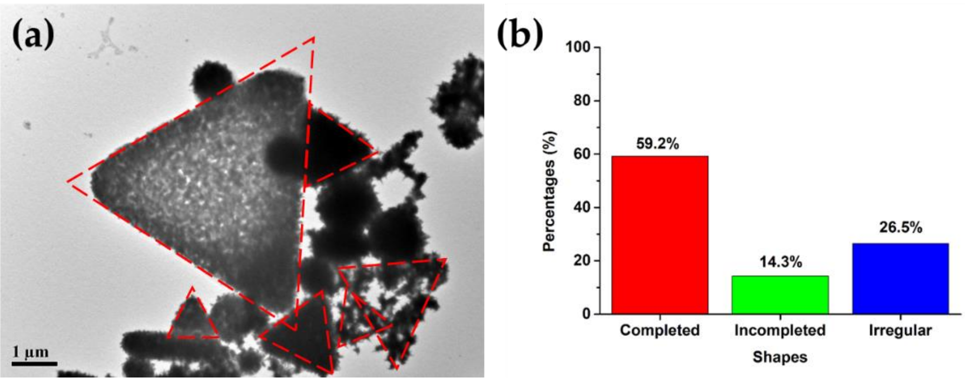

Figure S9. TEM image and statistical data of Ag@AuCu trigonal nanoplates. The dashed triangles in (**a**) mark the Ag@AuCu nanoplates with completed trigonal shape. The histogram in (**b**) show the percentages of three kinds of Ag@AuCu nanostructures: Completed - nanoplates with a clear trigonal boundary (both porous and solid nanoplates); Incompleted - incomplete shapes, like wires and trapezoids; Irregular - nanostructures with irregular shapes.

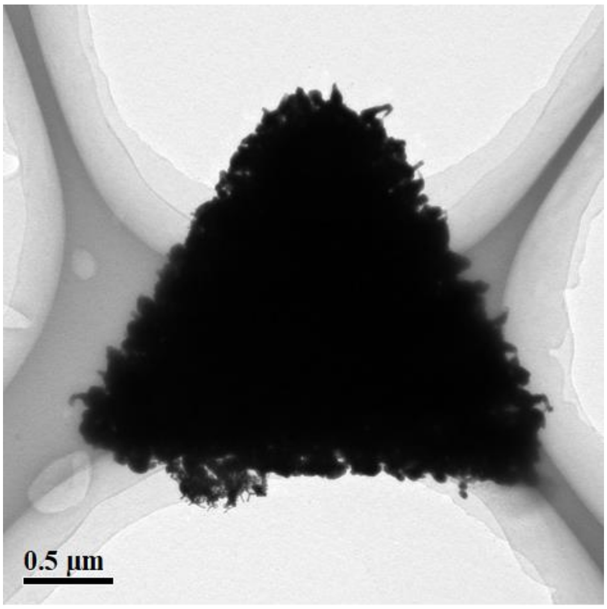

Figure S10. Growth of solid trigonal nanoplate of the Ag@AuCu nanostructures based on the AuCu alloy nanocrystals with an Au:Cu ratio of 0.33 using another method.

References

17. He, R.; Wang, Y.C.; Wang, X.; Wang, Z.; Liu, G.; Zhou, W.; Wen, L.; Li, Q.; Wang, X.; Chen, X.; Zeng, J.; Hou, J.G. Facile synthesis of pentacle gold–copper alloy nanocrystals and their plasmonic and catalytic properties. *Nat. Commun.* **2014**, 5, 4327. [CrossRef]
